# Recurrent arterial thrombosis in a patient with Fabry disease: case report

**DOI:** 10.1590/1677-5449.190096

**Published:** 2020-03-10

**Authors:** Altino Ono Moraes, Tiago Francisco Meleiro Zubiolo, Augusto Felipe Bruchez Brito, Jessica Belentani, Juliano Fabrício Santos, Gisele Nayara dos Santos, Lóren Fontinhas Faccin, Luanna Gabarrão Silva

**Affiliations:** 1 Instituto de Molestias Vasculares – IMV, Maringá, PR, Brasil.; 2 Hospital Santa Rita de Maringá, Maringá, PR, Brasil.; 3 Faculdade Ingá, Departamento de Medicina, Maringá, PR, Brasil.

**Keywords:** Fabry disease, X chromosome, alpha-galactosidase

## Abstract

Fabry disease is a rare disease, defined as an X-linked lysosomal deposition disease that presents with multisystemic symptoms, including vascular impairment with thrombotic events. A 57-year-old female patient diagnosed with Fabry disease 11 years previously, presented with hyperhidrosis, hypoacusis, and angiokeratoma on the hands. Her previous pathological history included an episode of ischemic stroke before the age of 40 years and chronic acute thrombosis in the right lower limb, 1 year previously, which had been treated with stent angioplasty, with temporary improvement followed by recent relapse of the condition. Thrombotic events fit the typical symptoms of Fabry disease and are caused by deposition of globotriaosylceramide in the vascular endothelium, constituting a prothrombotic state and explaining the recurrence of symptoms and arterial thrombosis in the lower limb.

## INTRODUCTION

Fabry disease (FD) is a lysosomal deposition disease caused by mutations of the GLA gene on chromosome X (Xq22.1).^1^^,^ 2 Incidence is 1:117,000 in the general population and it causes reduced or absent activity of the enzyme lysosomal alpha-galactosidase A (AGAL),^3^^,^ 4 which results in progressive build-up of globotriaosylceramide (Gb3) in the cells of a variety of tissues.^1^^,^ 2. Fabry disease presents with multisystemic signs and symptoms, including angiokeratoma, acroparaesthesia, diaphoresis abnormalities, cornea verticillata, chronic or episodic pain, cardiovascular, cerebrovascular, and renal disorders, such as cardiomyopathy, arrhythmia, and stroke, and proteinuria, imposing limited life expectancy.^1^^-^ 6 Angiokeratoma is the most common cutaneous manifestation, primarily seen in women and located on the trunk and limbs. Hypohidrosis is the most common sign reported by patients in general, while hyperhidrosis is less common, although more prevalent in women.^4^ Pain is possibly caused by deposition of Gb3 in dorsal root ganglia and sympathetic ganglia, or by small fiber neuropathy, which is also associated with failure to sense temperature. Episodic pain, known as “Fabry crises”, generally initiates in the extremities and radiates proximally, and can be triggered by exercise, diseases, temperature changes, or other physical and emotional stresses.^1^^,^ 4 Progressive accumulation of Gb3 in vascular endothelial cells transforms the endothelial surface into a procoagulatory and proinflammatory state, playing a critical role in pathogenesis and progression of thrombotic events,^3^^,^ 6 which have an incidence of 15% in people with FD.^3^ Vascular involvement is complex in these patients, affecting small arteries and with diffuse compromise of the arterial and venous systems. Storage of Gb3 in the arterial territory and subsequent proliferation of smooth muscle cells are considered the first manifestations of vascular involvement.^3^^-^ 5 As a result, people with FD are predisposed to premature arterial thrombotic complications and the objective of this article is to report a case that exemplifies this situation.

## CASE DESCRIPTION

**Figure 1:**
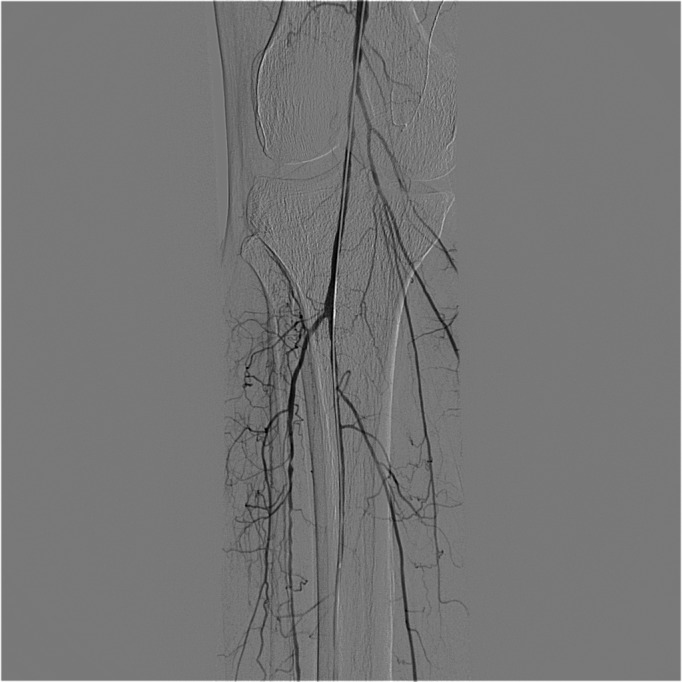
Arteriography showing critical stenosis of the popliteal artery, distal of the stent.

**Figure 2:**
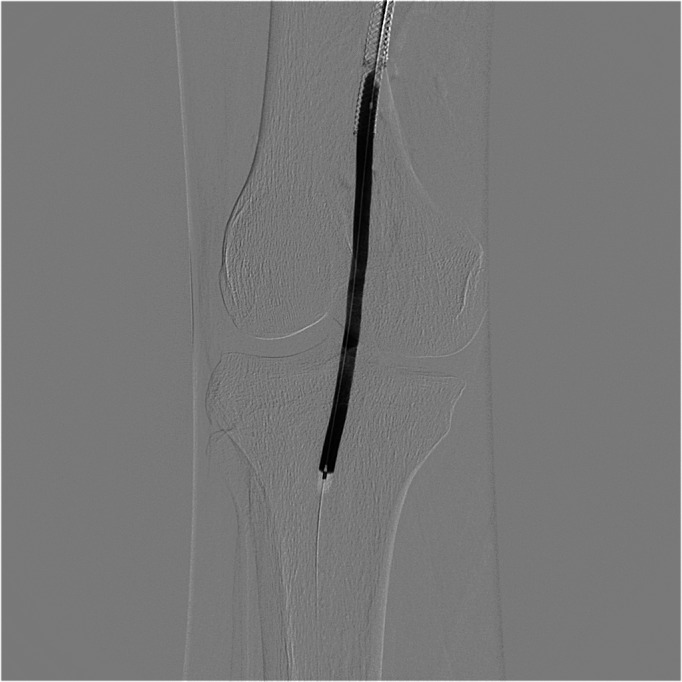
Angioplasty of the popliteal artery using drug-eluting balloon.

**Figure 3:**
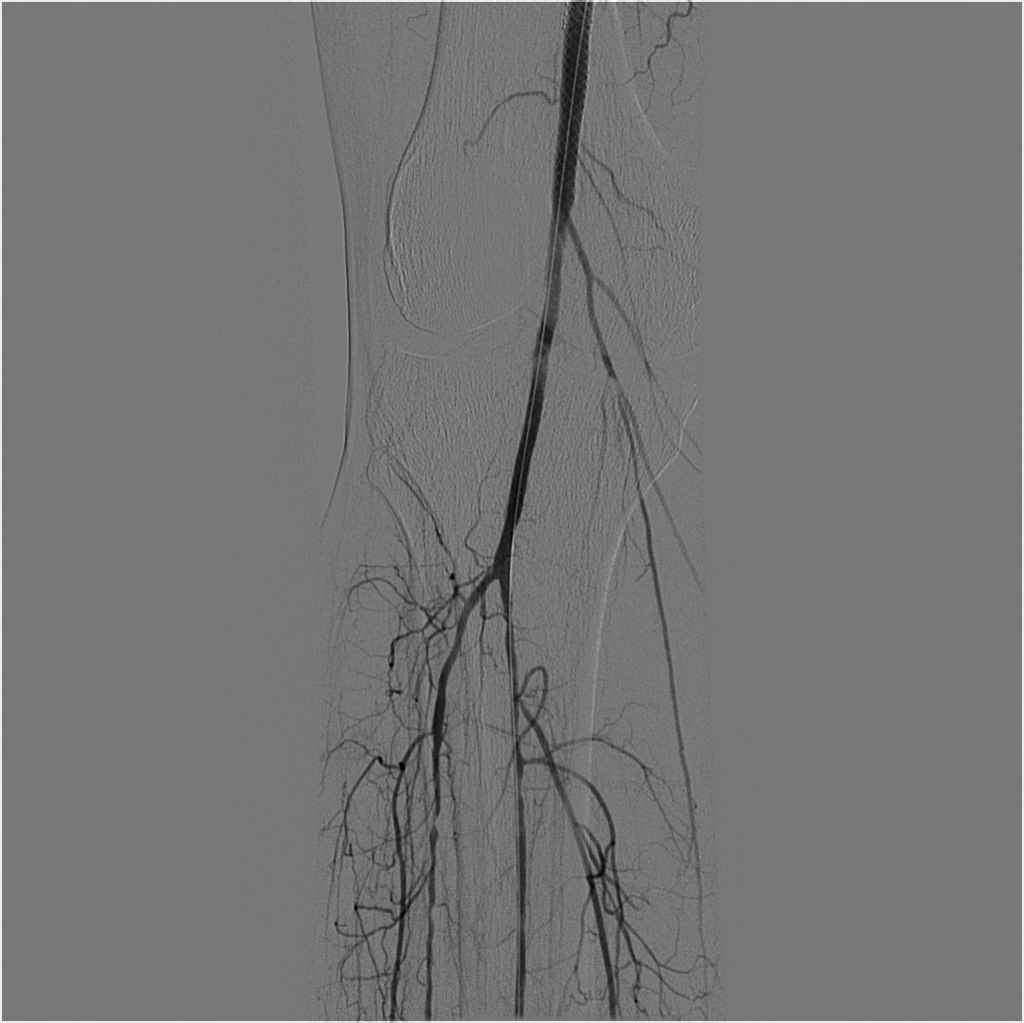
Final result after angioplasty of the popliteal artery using a drug-eluting balloon.

**Figure 4:**
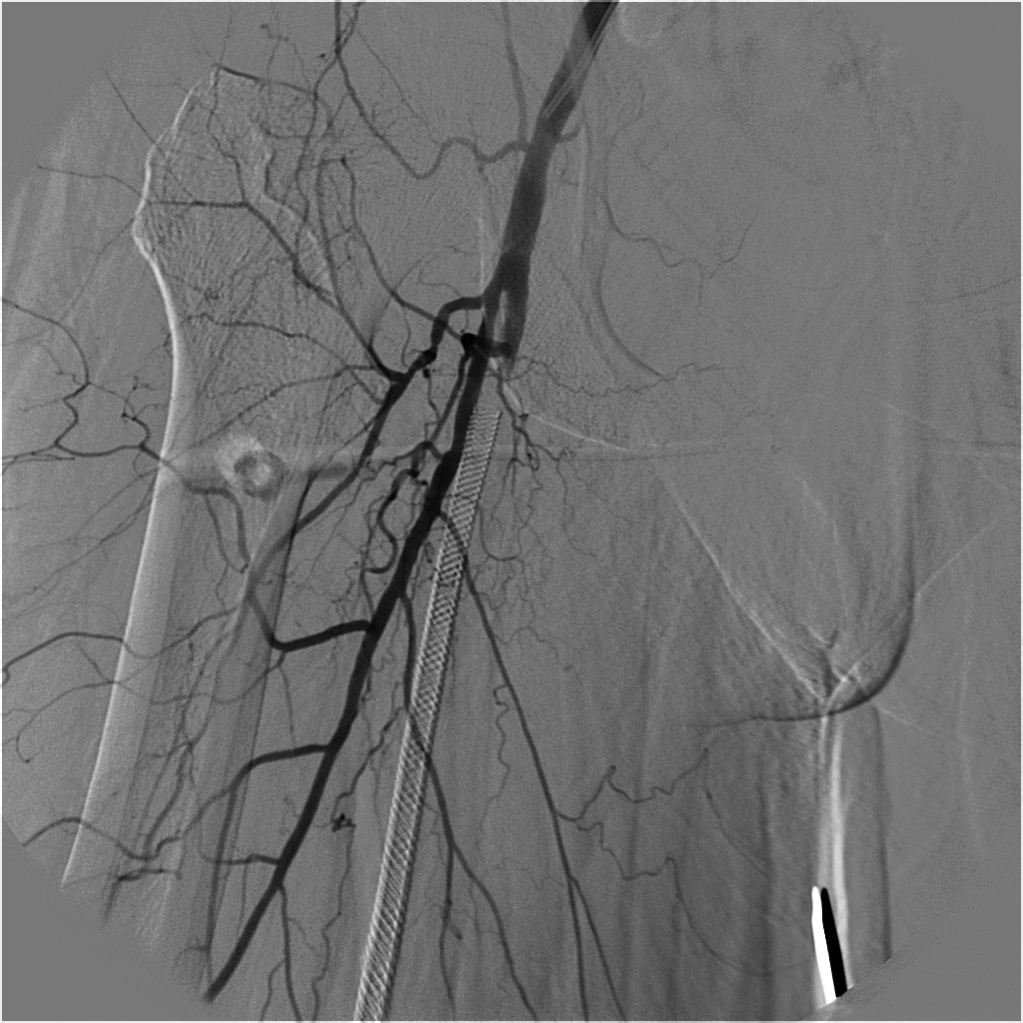
Arteriography showing occlusion of the superficial femoral artery in a proximal segment.

**Figure 5:**
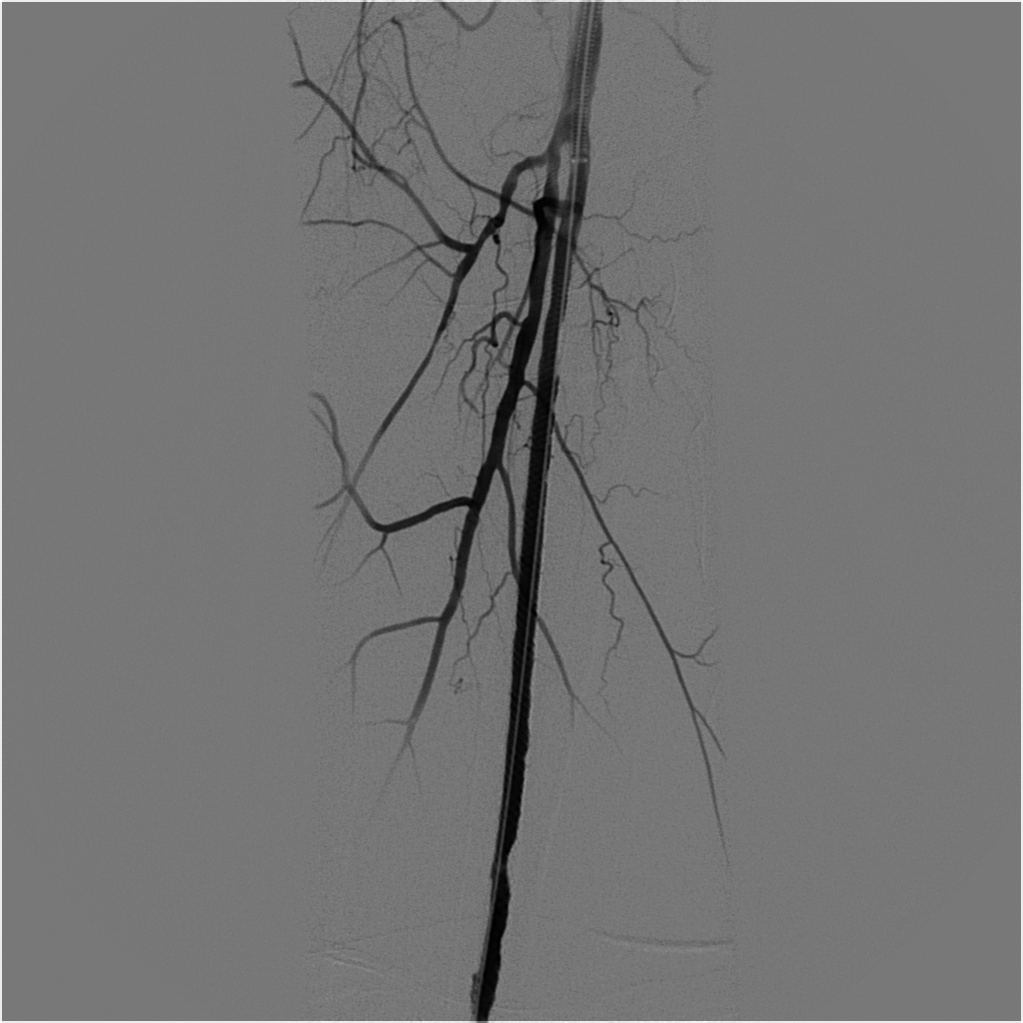
Arteriography after infusion of thrombolytic alteplase over 24 hours.

**Figure 6:**
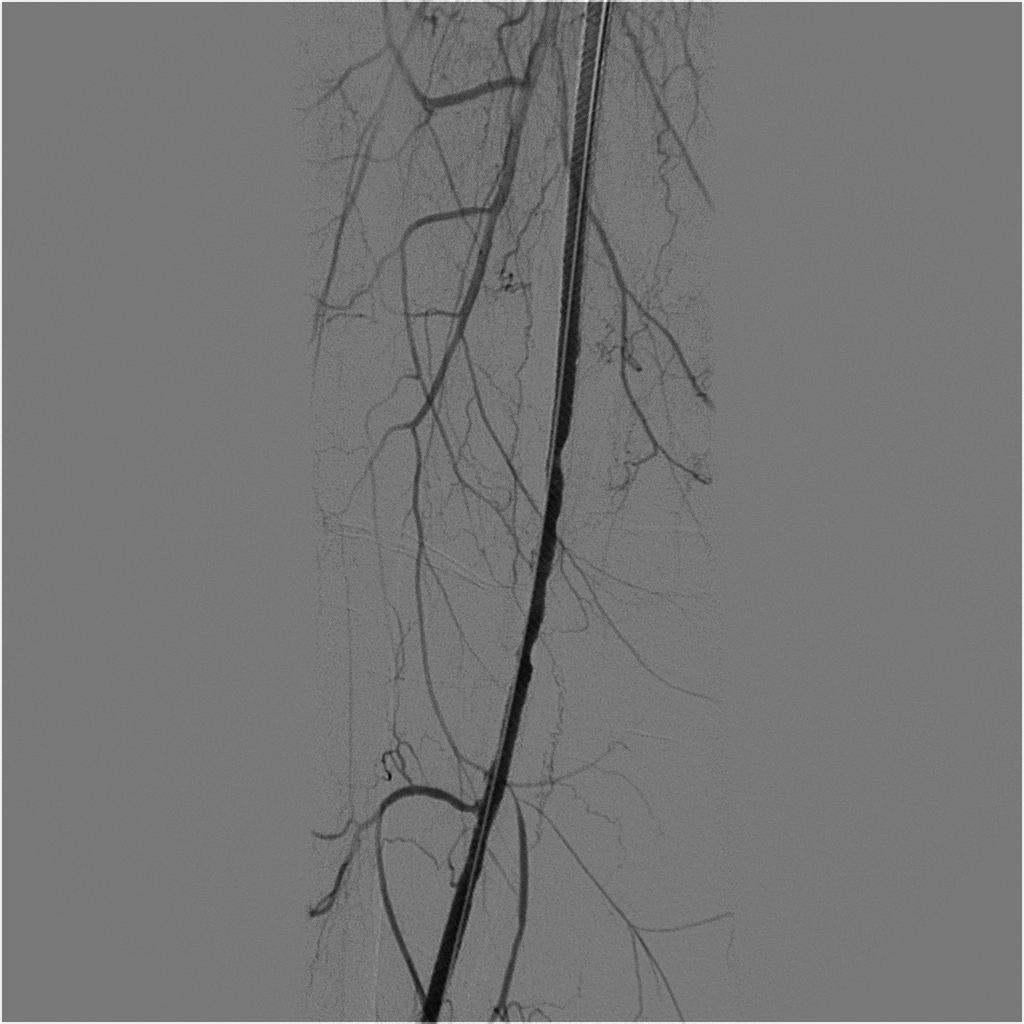
Final result after angioplasty with stenting of the superficial femoral artery.

**Figure 7:**
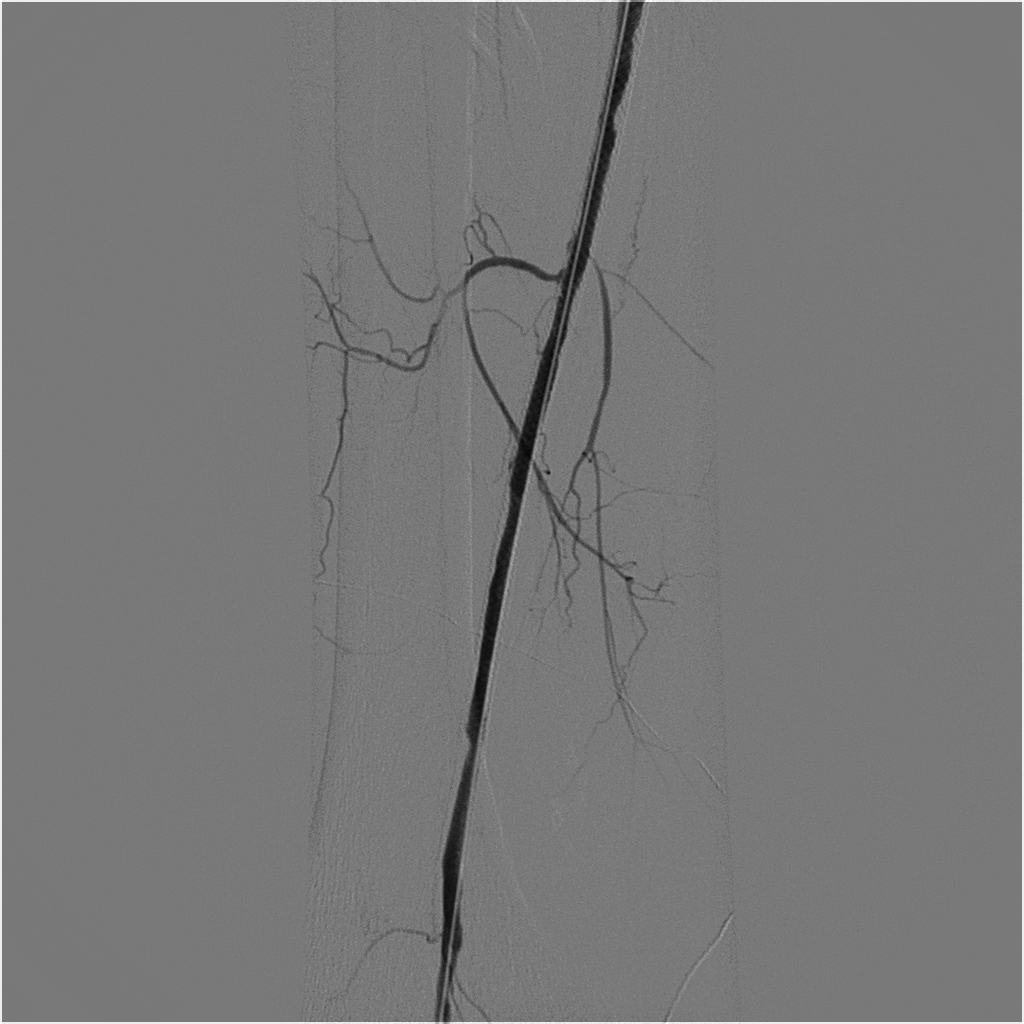
Control arteriography showing the femoropopliteal area.

The patient was a 57-year-old female who had been diagnosed with FD in 2007, confirmed by genetic tests that detected an abnormality of the GLA gene with the p.R342Q variant. She was a smoker, had systemic arterial hypertension, and was free from renal and cardiac complications. She had hyperhidrosis, hypoacusia, and angiokeratoma on the hands. She stated that she had had no other symptoms during the course of the disease. Her surgical history included three caesarean deliveries, varicose veins surgery, blepharoplasty, lipoaspiration, and mamoplasty. She was one of nine siblings, five of whom had been diagnosed with the disease (two males and three females), including the patient. Her mother, who had not been diagnosed, and both brothers suffered renal failure around the fourth decade of life. The patient had had an ischemic stroke episode at 40 years of age, without sequelae. Two years previously, she had complained of onset of pain at rest in the right lower limb, had undergone angiography of the limb and had been treated with percutaneous transluminal angioplasty with placement of a Supera Abbott® stent in the right superficial femoral artery (SFA), which was a technical success and resulted in resolution of the pain. The patient was put on double platelet antiaggregation with 100 mg acetylsalicylic acid (ASA) and 75mg clopidogrel and control color Doppler ultrasonography (CDUS) was conducted at 3-month intervals, showing that the stent was patent until, at 9 months of follow-up, the patient began to complain of intermittent claudication of the right lower limb after walking 50 meters. Angiotomography was conducted, showing the stent was patent, but revealing critical stenosis of the distal right popliteal artery (Figure 1). During the second procedure, we only conducted angioplasty with a Freeway Eurocor® drug-eluting balloon, with excellent results (Figures 2 and 3). After the procedure, we put the patient on 20 mg/day rivaroxaban and 100 mg/day ASA. After 12 months of follow-up after the second intervention, the patient presented with acute pain and paresthesia in the right leg and foot. She underwent urgent arteriography, which showed proximal occlusion of the right SFA (Figure 4). A Fountain Merit® catheter was fitted and 50 mg of alteplase was administered by continuous infusion with an infusion pump over 24 hours. After the infusion, the patient was taken to the hemodynamic room for arteriography, which demonstrated complete patency of the SFA and segment 1 of the right popliteal artery (Figure 5) and with 90% stenosis of segment 2 of the right popliteal artery. The decision was taken to deploy another Supera® stent and perform angioplasty, with excellent results and total reperfusion of the arterial tree (Figures 6 and 7). Rivaroxaban was reintroduced at 20 mg/day and the patient is in follow-up with CDUS at 3-month intervals. This patient has certain symptoms that are not typical of the disease, such as hyperhidrosis. The thrombotic events are the result of Gb3 deposition in the vascular endothelium, constituting a prothrombotic state that explains the recurrence of symptoms and arterial thrombosis in the lower limb.

## DISCUSSION

Fabry disease is hereditary and is linked to the X chromosome, meaning that men have a severe form of the disease and transmit it to all of their daughters, but not to their sons. In the case described here, the patient was female and had a less severe form of FD caused by random inactivation of one of the X chromosomes (the Lyon hypothesis)^2^ with manifestations such as those exhibited by this patient: angiokeratoma, intermittent claudication in response to minimal effort, prior ischemic stroke, and arterial thrombosis of a lower limb. However, the patient also exhibited certain signs and symptoms that are not typical of the disease, such as hyperhidrosis rather than hypohidrosis.

Although there is little literature on vascular involvement in FD, clinical and experimental evidence suggest that the process of infiltrative changes to the artery wall that lead to ischemia may differ from those observed in classic atherosclerosis, and FD-specific atherosclerosis is more diffuse and has a different composition to plaques, in addition to primarily involving small penetrating arterial vessels. As already mentioned, build-up of Gb3 leads to endothelial dysfunction and acceleration of the atherosclerotic process, caused by elevated levels of myeloperoxidase and reactive oxygen species. It is worth pointing out that when thrombophilias are also present, in particular factor V Leiden, severity of signs and symptoms is increased and events occur earlier.^7^

Many drug-based treatments are being studied; but agalsidase enzyme appears to be the most promising approach, treating the deficiency that causes disease, and should be initiated at onset of the first vascular events, thus avoiding late treatment and increased risk of amputations and chronic kidney disease requiring dialysis. This medication is already registered for use in Brazil under the name fabrazyme (Genzyme® – Sanofi Company), but it is expensive.^8^

The patient in this case report had intermittent claudication with onset in adulthood that relapsed after surgery and drug treatment, which can be explained by deposition of glycosphingolipids such as Gb3 in the vascular endothelium and visceral and nervous system tissues, which is also related to another clinical finding in this patient - stroke.^1^ With regard to the patient’s family history, there was symptomatology compatible with FD in two brothers, in both cases with severe, fatal manifestations. The osteomuscular, cardiopulmonary, renal, gastrointestinal, dermatological, and vascular complications in FD patients cause morbidity and mortality. In view of this, practitioners should be alert to the symptoms and institute treatment rapidly, as described, in order to avert sequelae that have a negative impact on patient quality of life, such as major limb amputations. As illustrated by the explanations detailed above, it can be concluded that FD is a serious disease that can cause permanently incapacitating complications, but which can be treated with drugs and surgery, improving patient prognosis.
