# Supplementary material for: Recurrent arterial thrombosis in a patient with Fabry disease: case report
Source: J Vasc Bras. 2020 Mar 10;19:e20190096. doi: 10.1590/1677-5449.190096 (PMC7244207; doi:10.1590/1677-5449.190096)
Supplement: Translation

# Translation: Trombose arterial recorrente em paciente com doença de Fabry: relato de caso

**Authors:** Altino Ono Moraes, Tiago Francisco Meleiro Zubiolo, Augusto Felipe Bruchez Brito, Jessica Belentani, Juliano Fabrício Santos, Gisele Nayara dos Santos, Lóren Fontinhas Faccin, Luanna Gabarrão Silva

**Affiliations:** 1 Instituto de Molestias Vasculares – IMV, Maringá, PR, Brasil.; 2 Hospital Santa Rita de Maringá, Maringá, PR, Brasil.; 3 Faculdade Ingá, Departamento de Medicina, Maringá, PR, Brasil.

**Keywords:** doença de Fabry, cromossomo X, alfa-galactosidase

## Abstract

A doença de Fabry é definida como uma doença rara de depósito lisossomal ligada ao cromossomo X que apresenta sintomas multissistêmicos, incluindo comprometimento vascular com eventos trombóticos. Paciente do sexo feminino, 57 anos, com diagnóstico de doença de Fabry há 11 anos, apresentava hiperidrose, hipoacusia e angioqueratoma nas mãos. Na história patológica pregressa, relatou episódio de acidente vascular encefálico isquêmico prévio aos 40 anos de idade e trombose arterial crônica agudizada em membro inferior direito há 1 ano, a qual foi tratada por meio de angioplastia com uso de stent, apresentando melhora temporária e recente recidiva do quadro. Os eventos trombóticos se enquadram nos sintomas típicos da doença de Fabry, e são resultantes do depósito de globotriaosilceramida no endotélio vascular, implicando em um estado pró-trombótico, justificando a reincidência dos sintomas e da trombose arterial em membro inferior.

## INTRODUÇÃO

A doença de Fabry (DF) é uma doença de depósito lisossomal causada por mutações no gene GLA no cromossomo X (Xq22.1)^1^^,^ 2. Sua incidência é de 1:117.000 na população geral e resulta em atividade diminuída ou ausente da enzima lisossômica alfa-galactosidase A (AGAL)^3^^,^ 4, levando ao acúmulo progressivo de globotriaosilceramida (Gb3) nas células de vários tecidos^1^^,^ 2. A DF apresenta sinais e sintomas multissistêmicos, incluindo angioqueratoma, acroparestesia, anormalidades da sudorese, córnea verticilata, dor crônica ou episódica, distúrbios cardiovasculares, cerebrovasculares e renais, como cardiomiopatia, arritmia, acidente vascular encefálico (AVE) e proteinúria, que acarretam uma expectativa de vida limitada^1^^-^ 6. O angioqueratoma é a manifestação cutânea mais comum, sendo encontrado principalmente nas mulheres e estando localizado no tronco e nos membros. A hipoidrose é o sinal mais comum relatado pelos pacientes em geral, sendo a hiperidrose menos comum, porém mais prevalente em mulheres^4^. A dor é possivelmente causada pela deposição de Gb3 nos gânglios da raiz dorsal e nos gânglios simpáticos, ou por neuropatia de pequenas fibras, que também está associada à falta da percepção da temperatura. A dor episódica na DF, denominada “crise de Fabry”, geralmente começa nas extremidades e irradia-se proximalmente, podendo ser desencadeada por exercício, doenças, mudanças de temperatura, ou outros estresses físicos e emocionais^1^^,^ 4. O acúmulo progressivo de Gb3 nas células endoteliais vasculares resulta na conversão da superfície do endotélio em estado pró-coagulante e pró-inflamatório, desempenhando um papel crítico na patogênese e na progressão de acidentes trombóticos^3^^,^ 6, que apresentam uma incidência de 15% em indivíduos com DF^3^. O comprometimento vascular nesses pacientes é complexo, apresentando envolvimento de pequenas artérias, além do comprometimento difuso dos sistemas arterial e venoso. O armazenamento de Gb3 no território arterial e a subsequente proliferação de células musculares lisas são considerados as primeiras manifestações de envolvimento vascular^3^^-^ 5. Portanto, os indivíduos com DF estão predispostos a complicações trombóticas arteriais prematuras, e este trabalho tem como objetivo relatar um caso que exemplifica essa situação.

## DESCRIÇÃO DO CASO

Paciente do sexo feminino, 57 anos de idade, com diagnóstico de DF desde 2007, com confirmação a partir de estudo genético apresentando alteração do gene GLA, com presença de variante p.R342Q. Tabagista, portadora de hipertensão arterial sistêmica, sem complicações renais e cardíacas. Apresenta hiperidrose, hipoacusia e angioqueratoma nas mãos. Relata ausência de outros sintomas durante todo o curso da doença. Possui antecedentes cirúrgicos de três cesáreas, cirurgia de varizes, blefaroplastia, lipoaspiração e mamoplastia. Pertencente a uma família de nove irmãos, sendo que cinco desses foram diagnosticados com a doença (dois homens e três mulheres), incluindo a paciente. Sua mãe, sem diagnóstico, e os dois irmãos apresentaram insuficiência renal por volta da quarta década de vida. Teve um episódio de AVE isquêmico aos 40 anos de idade, sem sequelas. Há dois anos, apresentou quadro de dor ao repouso em membro inferior direito, sendo submetida a angiografia do membro seguida de angioplastia transluminal percutânea com implante de stent Supera Abbott® em artéria femoral superficial (AFS) direita, apresentando sucesso técnico e desaparecimento da dor. Foi feito controle com eco-Doppler colorido (EDC) de membros em intervalos de 3 meses, que mostrava perviedade do stent usando terapia de dupla antiagregação plaquetária com ácido acetilsalicílico (AAS) 100 mg e clopidogrel 75 mg, até que, após 9 meses de seguimento, a paciente começou a referir claudicação intermitente de membro inferior direito para 50 metros. Foi então realizada angiotomografia, que mostrou stent pérvio e estenose crítica de artéria poplítea direita distal (Figura 1). No segundo procedimento, fizemos apenas angioplastia com balão farmacológico Freeway Eurocor®, com resultado excelente (Figuras 2 e 3). Após o procedimento, optamos por iniciar **r**ivaroxabana 20 mg/dia e AAS 100 mg/dia. Aos 12 meses de seguimento, após a segunda intervenção, a paciente apresentou dor aguda e parestesia em perna e pé direitos. Foi submetida a arteriografia de urgência, que mostrou oclusão proximal de AFS direita (Figura 4), sendo então implantado cateter Fountain Merit® e feita infusão continua, em bomba infusora, de 50 mg de alteplase por 24 horas. Após a infusão, a paciente foi levada à hemodinâmica para realização de nova arteriografia, que demonstrou abertura total da AFS e do segmento 1 da artéria poplítea direita (Figura 5) e estenose de 90% do segmento 2 da artéria poplítea direita . Optou-se então por implante de novo stent Supera® e angioplastia, com ótimo resultado e reperfusão total da árvore arterial (Figuras 6 e 7). Foi reintroduzida rivaroxabana 20 mg/dia, e está sendo realizado acompanhamento com EDC em intervalos de 3 meses. A paciente em questão apresenta alguns sintomas não típicos da doença, como hiperidrose. Os eventos trombóticos são resultantes do depósito de Gb3 no endotélio vascular, implicando em um estado pró-trombótico e justificando a reincidência dos sintomas e da trombose arterial em membro inferior.

**Figura 1:** Arteriografia evidenciando estenose crítica em artéria poplítea distal ao stent.

**Figura 2:** Angioplastia de artéria poplítea utilizando balão farmacológico.

**Figura 3:** Resultado final pós-angioplastia de artéria poplítea utilizando balão farmacológico.

**Figura 4:** Arteriografia mostrando oclusão da artéria femoral superficial em segmento proximal.

**Figura 5:** Arteriografia após infusão do trombolítico alteplase durante 24 horas.

**Figura 6:** Resultado final pós-angioplastia com stent em artéria femoral superficial.

**Figura 7:** Arteriografia de controle do território femoropoplíteo.

## DISCUSSÃO

A DF tem caráter hereditário e é relacionada ao cromossomo X; sendo assim, os homens apresentam a doença de forma grave e transmitem-na a todas as filhas, mas não aos filhos. No caso em questão, trata-se de uma paciente do sexo feminino, em que a DF se apresenta de forma menos grave, devido à inativação aleatória de um dos cromossomos X (hipótese de Lyon)^2^, e tem manifestações como as apresentadas pela paciente: angioqueratoma, claudicação intermitente aos mínimos esforços, AVE isquêmico prévio e a trombose arterial de membro inferior. Mas a paciente aqui relatada também apresenta alguns sinais e sintomas não típicos da doença, como hiperhidrose em vez de hipoidrose.

Embora o envolvimento vascular na DF tenha escassa literatura, evidências clínicas e experimentais sugerem que o processo de mudanças infiltrativas na parede arterial levando à isquemia pode diferir daquele observado na aterosclerose clássica, sendo as ateroscleroses específicas da DF mais difusas e com composição diferente das placas, além de envolverem principalmente os pequenos vasos arteriais penetrantes. Com já comentado, com o acúmulo de Gb3 ocorre disfunção endotelial e aceleração do processo aterosclerótico, causadas pelo aumento nos níveis de mieloperoxidase e de espécies reativas de oxigênio. Ressalta-se que a associação com trombofilias, principalmente com a presença do fator V de Leiden, leva a uma maior gravidade dos sinais e sintomas e à ocorrência de eventos mais precoces^7^.

Diversos tratamentos farmacológicos estão em estudo; porém, o uso da enzima agalsidase se mostrou mais promissor para suprir a deficiência causadora da doença, devendo ser iniciado com os primeiros eventos vasculares, evitando início tardio e maiores riscos de amputações e de doença renal crônica dialítica. Trata-se de um medicamento já registrado para uso no território nacional com o nome de fabrazyme (Genzyme® – Sanofi Company), mas tem custo muito elevado^8^. A paciente deste relato apresenta claudicação intermitente iniciada na idade adulta, recidivada após cirurgia e uso de medicamentos, fato que pode ser explicado pelo depósito dos glicoesfingolipídeos como a Gb3 no endotélio vascular, nos tecidos viscerais e do sistema nervoso, que também se relaciona a outro achado clínico da paciente, o AVE^1^. Quanto ao histórico familiar da paciente, há sintomatologia compatível com a DF em dois irmãos do sexo masculino, sendo as manifestações graves e fatais em ambos. As complicações osteomusculares, cardiopulmonares, renais, gastrointestinais, dermatológicas e vasculares implicam na morbimortalidade dos portadores da DF. Sendo assim, deve-se atentar aos sintomas e fazer o tratamento rapidamente, conforme relatado, a fim de evitar sequelas que afetem negativamente a qualidade de vida do paciente, como uma amputação maior de membro. Portanto, por meio das explanações aqui apresentadas, conclui-se que a DF é uma doença grave, com possíveis complicações incapacitantes permanentes, mas com possibilidade de tratamento medicamentoso e cirúrgico, dando assim um prognóstico melhor aos pacientes.
